# TRPV4 calcium entry and surface expression attenuated by inhibition of myosin light chain kinase in rat pulmonary microvascular endothelial cells

**DOI:** 10.1002/phy2.121

**Published:** 2013-10-27

**Authors:** James C Parker, Masahiro Hashizumi, Sarah V Kelly, Michael Francis, Marc Mouner, Angela L Meyer, Mary I Townsley, Songwei Wu, Donna L Cioffi, Mark S Taylor

**Affiliations:** Department of Physiology and Center for Lung Biology, College of Medicine, University of South AlabamaMobile, Alabama, 36688

**Keywords:** Biotinylation, dynasore, ECIS, Ventilator-induced lung injury, vesicles

## Abstract

In previous studies, blockade or gene deletion of either myosin light chain kinase (MLCK) or the mechanogated transient receptor potential vanilloid 4 (TRPV4) channel attenuated mechanical lung injury. To determine their effects on calcium entry, rat pulmonary microvascular endothelial cells (RPMVEC) were labeled with fluo-4 and calcium entry initiated with the TRPV4 agonist, 4α-phorbol 12, 13-didecanoate (4αPDD). Mean calcium transients peaked at ∼25 sec and persisted ∼500 sec. The 4αPDD response was essentially abolished in calcium-free media, or after pretreatment with the MLCK inhibitor, ML-7. ML-7 also attenuated the 4αPDD-induced inward calcium current measured directly using whole-cell patch clamp. Pretreatment with dynasore, an inhibitor of dynamin produced an initial calcium transient followed by a 4αPDD transient of unchanged peak intensity. Automated averaging of areas under the curve (AUC) of calcium transients in individual cells indicated total calcium activity with a relationship between treatment groups of ML-7 + 4αPDD < 4αPDD only < dynasore + 4αPDD. Measurement of biotinylated surface TRPV4 protein indicated a significant reduction after ML-7 pretreatment, but no significant change with dynasore treatment. RPMVEC monolayer electrical resistances were decreased by only 3% with 10 μmol/L 4αPDD and the response was dose-related. Dynasore alone produced a 29% decrease in resistance, but neither ML-7 nor dynasore affected the subsequent 4αPDD resistance response. These studies suggest that MLCK may inhibit mechanogated calcium responses through reduced surface expression of stretch activated TRPV4 channels in the plasma membrane.

## Introduction

Ventilator-induced lung injury (VILI) is a significant contributor to the mortality in the adult respiratory distress syndrome (ARDS). In a large-scale clinical study, a decrease in tidal volume from 12 to 6 mL/kg resulted in a 22% increase in survival in ARDS patients (Brower et al. 2000). Lung overdistention activates multiple inflammatory pathways, many of which are common to multiple permeability inducing agonists (Dreyfuss and Saumon 1998; Parker et al. 2006; dos Santos and Slutsky 2006; Birukov 2009). However, the acute increase in pulmonary vascular permeability associated with lung overdistention appears to be initiated in large part by calcium influx through stretch-activated cation channels rather than receptor operated channels (Parker et al. 1998). These stretch-activated channels have recently been identified as the transient receptor potential vanilloid 4 (TRPV4) channel because inhibition or gene deletion of TRPV4 attenuates lung vascular permeability increases during high-pressure ventilation (Hamanaka et al. 2007). Although several phosphorylation and binding sites have been identified at the molecular level on the TRPV4 channel, which may alter its sensitivity (Nilius et al. 2003; Watanabe et al. 2003; Everaerts et al. 2010), the regulators of mechanical sensitivity in intact lungs remain a mystery and currently there are no accepted pharmacologic treatments available for preventing VILI (Parker and Townsley 2011).

A critical step in initiating increased permeability and inflammation in the lungs after both mechanical stress and sepsis is also mediated by an endothelial myosin light chain kinase (MLCK) (Mirzapoiazova et al. 2011). In previous isolated lung studies, we demonstrated that inhibition of MLCK attenuated the increased permeability effects of high airway pressure ventilation (Parker 2000). Subsequent studies demonstrated attenuation of both ventilator-induced and sepsis-induced lung injuries in mice with a genetic deletion of nonmuscle MLCK, or after the suppression of MLCK activity using specific inhibitory peptides or specific siRNA (Wainwright et al. 2003; Mirzapoiazova et al. 2011). Previous studies have established that increased intracellular calcium entry, or store release, causes activation of calmodulin and activation of MLCK with phosphorylation of myosin light chains, which can increase cytoskeletal tension and widening intercellular junctions to increase vascular permeability (Wysolmerski and Lagunoff 1991; Garcia and Schaphorst 1995; Garcia et al. 1999; Birukov et al. 2001; Dudek et al. 2010).

Inhibition of MLCK may also have a less appreciated impact on vascular permeability by reducing the calcium entry which initiates activation of these intracellular permeability mechanisms. For example, inhibition of MLCK greatly attenuated calcium entry in endothelial cells induced by shear stress, bradykinin and thapsigargin (Watanabe et al. 1998, 2001; Norwood et al. 2000). Reduced store operated calcium entry after MLCK inhibition was attributed to a decrease in vesicular shuttling of calcium permeable channels such as the transient receptor potential canonical channels, TRPC1 and TRPC4 to the cell membrane by microtubular motor proteins (Bauer and Stevens 2002; Wu et al. 2007). The constitutively active calcium channels, TRPC5 and TRPC6, have activity that is largely regulated by surface expression induced by vesicle traffic to and from the cell membrane (Bezzerides et al. 2004; Cayouette et al. 2004; Cayouette and Boulay 2007). In other studies, TRPV4 surface expression was enhanced when dynamin-mediated endocytosis was blocked (Cuajungco et al. 2006). Furthermore, vesicle exocytosis was also found to be dependent upon myosin activity (Watanabe et al. 2005; Eichler et al. 2006). However, the role of vesicle transport in TRPV4 activity in lung endothelial cells and the involvement of MLCK in this process have not been extensively investigated.

In this study, we measured calcium entry transients and monolayer electrical resistance in rat pulmonary microvascular endothelial cells (RPMVEC) after activation of TRPV4 using the TRPV4 agonist, 4α-phorbol 12, 13-didecanoate (4αPDD). Calcium transients in RPMVECs were measured using fluorescent microscopy and whole-cell patch clamp methods. Increases in intracellular calcium in individual cells were also detected and statistically analyzed using the custom LCPro analysis program (Francis et al. 2012), with and without pretreatment with ML-7 or dynasore, the respective inhibitors of MLCK and dynamin. Surface expression of TRPV4 protein was measured by biotinylation of surface proteins followed by Western blots with and without ML-7, or dynasore. Monolayer electrical impedances in these treatment groups were also measured using electrical cell-substate impedance sensing (ECIS).

## Methods

### Isolation and culture of rat lung endothelial cells

All experimental protocols were approved by the institutional Animal Care and Use Committee of the University of South Alabama, College of Medicine. RPMVECs were obtained from the Center for Lung Biology Cell Culture Lab where cells were cultured as previously described (Stevens and Thompson 1999), and characterized using SEM, uptake of l,l′-dioctadecyl-3, 3,3′, 3′-tetramethylin-docarbocyanine-labeled low-density lipoprotein (Dil-acetylated LDL), and a lectin-binding panel of *Helix pomatia, Griffonia simplicifolia,* and *Glycine max*. *Helix pomatia* is selective for RPMVEC whereas *Griffonia simplicifolia* and *Glycine max* are selective for rat pulmonary artery endothelial cells (RPAEC) (King et al. 2004). RPMVEC were grown to confluence in Dulbecco's Modified Eagle Medium (DMEM) with 10% fetal bovine serum (FBS).

### Drugs

4αPDD, ML-7, and dynasore hydrate were obtained from Sigma-Aldrich Corp., St. Louis, MO. Drugs were dissolved in dimethyl sulfoxide (DMSO) to make stock solutions to obtain target doses of 10 μmol/L of 4αPDD using 7 μL per mL final volume, 80 μmol/L of dynasore using 5 μL per mL final volume, and 1 μmol/L of ML-7 using 5 μL per mL final volume. ML-7, is a cell-permeable inhibitor of MLCK (Ki = 300 nmol/L), which also inhibits PKA (Ki = 21 mmol/L) and PKC (Ki = 42 mmol/L) at much higher concentrations than used in this study. Volumes of stock solution were adjusted depending upon the total volume of the well or culture dish and desired dose. Comparable volumes of DMSO were added to the control or baseline groups of each study.

### Immunocytochemistry

RPMVEC were seeded onto glass coverslips, grown to confluence, then washed and briefly fixed with 4% paraformaldehyde. Fixed cells were permeabilized and blocked with 0.1% Triton-×100 and 5% bovine serum albumin in phosphate-buffered saline (PBS), respectively. Permeabilized cells were costained for TRPV4 using a primary anti-TRPV4 antibody (1:200, Alomone Labs, Jerusalem, Israel) and an Alexa-fluor 594-conjugated secondary antibody (1:500, Invitrogen, Grand Island, NY) and focal adhesion kinase (FAK) using a primary anti-FAK antibody preconjugated with Alexa-fluor 488 (1:50, Invitrogen). Prior to imaging, nuclei were briefly stained with 4′,6-diamidino-2-phenylindole (DAPI) (1:6000, Invitrogen). Confocal images at the basal cell borders were obtained using a 60× objective (Nikon A1, Melville, NY).

### Confocal microscopy

RPMVEC were grown to confluence in coverslip chambers. Cells were loaded with a physiological saline solution (4-(2-hydroxyethyl)-1-piperazineethanesulfonic acid [HEPES], in mmol/L: 134 NaCl, 6 KCl, 1MgCl, 10 HEPES, 10 Glucose) containing fluo-4 fluorescent Ca^2+^ indicator dye (10 μmol/L) and Pluronic (0.03%) for 35 min at 25°C. After a 5-min wash and 20-min equilibration period, cells were mounted in a custom chamber and viewed on a Perkin Elmer (Santa Clara, CA) RS-3 spinning disk inverted confocal microscope. Excitation and emission wavelengths were 488 nm and 510 nm, respectively. Cells were challenged with 10 μmol/L 4αPDD in 2 mmol/L Ca^2+^ media, with or without pretreatment with 1 μmol/L ML-7, 80 μmol/L dynasore, or Ca^2+^-free media. An equivalent volume of DMSO alone did not induce a detectable calcium response. Fluorescence intensities were evaluated for the entire microscope field and in individual cells defined by single cell regions of interest (ROI) and recorded at 20× magnification with Perkin Elmer Ultraview software. Recordings at one frame per second were analyzed offline with ImageJ image analysis software and the LCPro subroutine to identify and analyze software identified region of interest (ROI) responses using our previously described automated ROI algorithm (Francis et al. 2012).

### Biotinylation and Western blotting

Cultured RPMVEC were grown to confluence and incubated with treatment drugs for 2 h at 37°C with 5% CO_2_ in media containing 10% FBS. Control or baseline groups were incubated with and equivalent volume of DMSO alone. Confluent monolayers were separated from the plates with cold PBS or mechanically detached to expose both apical and basal surface proteins. Plates were washed for 5 min in cold PBS containing calcium and 15 μL of 10 mmol/L biotin added. Cells were incubated for 1 h at 4°C then 30 μL of 100 mmol/L glycine added to stop the biotinylated in reaction. Cells were washed two times in cold PBS and centrifuged at 1000 rpm for 15 min. The supernatant was discarded and the pellet treated with 200 μL Hunter's lysis buffer, sonicated for 10 sec and centrifuged at 14,000 rpm for 15 min at 4°C. Supernatant proteins were collected and a Bradford assay performed to measure protein concentration. Supernatants were incubated overnight with Streptavidin beads to pull down the biotinylated proteins. The proteins were eluted by boiling with 2× Lamelli buffer for 5 min and centrifuged. Samples volume**s** were equalized and 25 μL per lane loaded on NuPage gels and run at 200 V with NuPage running buffer (Life Technologies, Grand Island, NY). Proteins were transferred to membranes using NuPage transfer buffer and membranes stained with Ponceau S red stain to check for equal loading. Membranes were blocked with 5% milk solution and incubated with primary antibody for the TRPV4 channel protein (Alomone) overnight at 4°C. Membranes were then incubated with goat anti-rabbit IgG peroxidase secondary antibodies for 1 h at room temperature, reacted with SuperSignal (Pierce, Thermo Fisher Scientific, Rockford, IL) luminol solution and photographed. Band intensities were measured using the UN-SCAN-IT analysis program.

### Patch-clamp electrophysiology

Whole-cell macroscopic currents were recorded using an EPC-9 amplifier (HEKA Elektronik, Bellmore, NY) as described previously (Wu et al. 2003). Data were acquired with Pulse/PulseFit software (HEKA) and filtered at 2.9 kHz. Voltage-dependent currents were corrected for linear leak and residual capacitance using an online subtraction paradigm. The extracellular bath solution contained (mmol/L) CaCl_2_ 10, tetraethylammonium chloride 110, CsCl 10, and HEPES 10 (pH 7.4, adjusted with tetraethylammonium hydroxide). Intracellular (pipette) solution contained (mmol/L) *N*-methyl-D-glucamine 130, EGTA (ethylene glycol tetra-acetic acid) 10, BAPTA (1,2-bis(o-aminophenoxy)ethane-N,N,N′,N′-tetraacetic acid) 5, HEPES 10, MgCl_2_ 6, CaCl_2_ 4, and Mg-ATP 2 (pH 7.2, adjusted with methane sulfonic acid). All solutions were adjusted to 290–300 mOsm with sucrose. Drugs were applied to the cells by a gravity-driven perfusion device, operated by a perfusion valve controller (model VC-6; Warner Instrument, Hamden, CT).

### ECIS analyses

Electrical resistance to an alternating current was measured across cell monolayers by using an ECIS system (Applied Biophysics, Troy, NY) (Schaphorst et al. 2003). Cells were grown to confluence on gold microelectrodes (10^−3^ cm^2^) (Applied Biophysics) connected to a phase-sensitive lock-in amplifier (model 5301A; EG&G Instruments, Princeton, NJ). After establishing a baseline resistance, drugs are added by withdrawal of 100 μL and replacing 100 μL with media containing either the appropriate drug doses or DMSO alone.

### Statistical analysis

All values are expressed as means ± SE. Groups were compared using a one-way analysis of variance (ANOVA) with repeated measures followed by a Student–Newman–Keuls posttest. Significant differences were determined where *P* < 0.05.

## Results

### Basilar localization of TRPV4 channels

**Figure 1:**
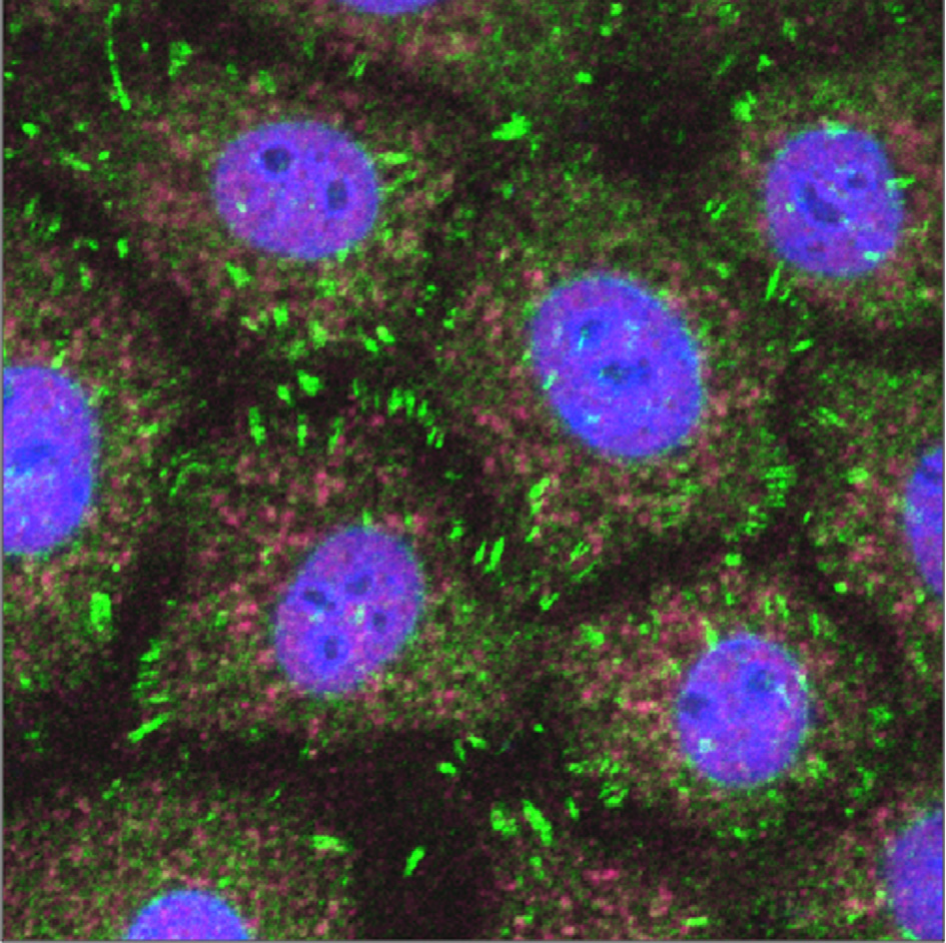
Confocal fluorescent micrographs showing localization of focal adhesion kinase (green) and TRPV4 channels (red) in basilar region of RPMVEC. Cell nuclei are shown in blue.

Figure 1 shows localization of FAK and TRPV4 in RPMVEC monolayers labeled with fluorescent antibodies against TRPV4 and FAK. TRPV4 (red) and FAK (green) were localized near the basal cell surface, although no colocalization of the two proteins was detected. Nuclei (blue) are labeled with DAPI. This figure is a confocal optical slice through the basal region of the monolayer as little label was detected in more apical regions. A basilar location of TRPV4 was further confirmed in biotinylation experiments where detectable amounts of TRPV4 were only recovered after separating the monolayer from the culture dish.

### Calcium responses to 4αPDD

**Figure 2:**
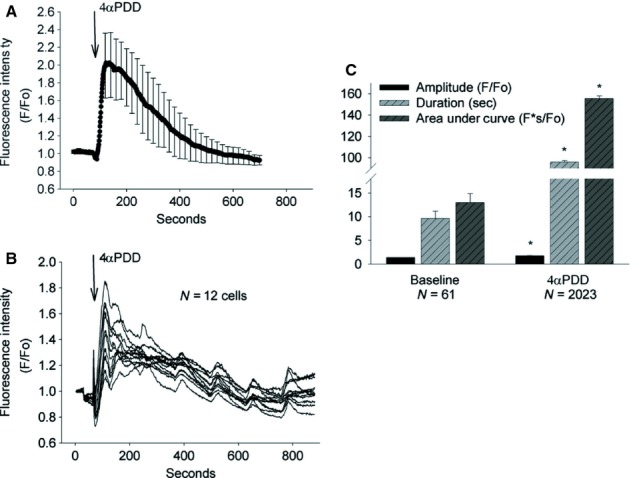
Intracellular calcium measured by fluo-4 fluorescence in response to 10 μmol 4αPDD. Shown are (A) mean fluorescent intensity (F/Fo) of four monolayers of RPMVEC; (B) fluorescent intensities of 12 individual cells within a monolayer; and (C) mean values for amplitude, duration, and AUC at half maximal amplitude in individual ROI determined by LCPro in four monolayers. The number of ROI are shown. *P < 0.05 versus baseline for the same parameter.

The intracellular calcium transients induced by the TRPV4 agonist, 4αPDD, were analyzed by measuring both the average fluorescent intensity of the RPMVEC monolayer, and fluorescent intensities of individual cells using both hand-drawn ROI and ROI in responding cells identified by the LCPro analysis program. Figure 2 indicates these 4αPDD calcium responses showing (1) the average fluorescence intensity of three monolayers, (2) activities of 12 individual ROI approximating the size of a single cells, and (3) statistical analysis of 2063 cells with responses reaching the threshold for detection by the LCPro software relative to amplitude (*F*/*F*o), curve duration at half maximal response (*S*), and area under curve (AUC = *F* × *S*/*F*o) above the half maximal response. The mean intensity increased approximately twofold, and peaked after approximately 40 sec with a total duration of ∼8 min. Continuous small calcium oscillations occurred even during baseline conditions in a heterogeneous manner with some ROIs reaching the threshold level for recording. Regional spreading of the calcium signal and some coordination is indicated by the synchronous waves in the last portion of Figure 2B. Activation with 4αPDD recruited calcium entry into most of the cells on the monolayer. The means for parameters calculated by the model were somewhat different than those derived from the mean of the three average field intensities due to weighting by the software of those ROI that responded to the treatment.

**Figure 3:**
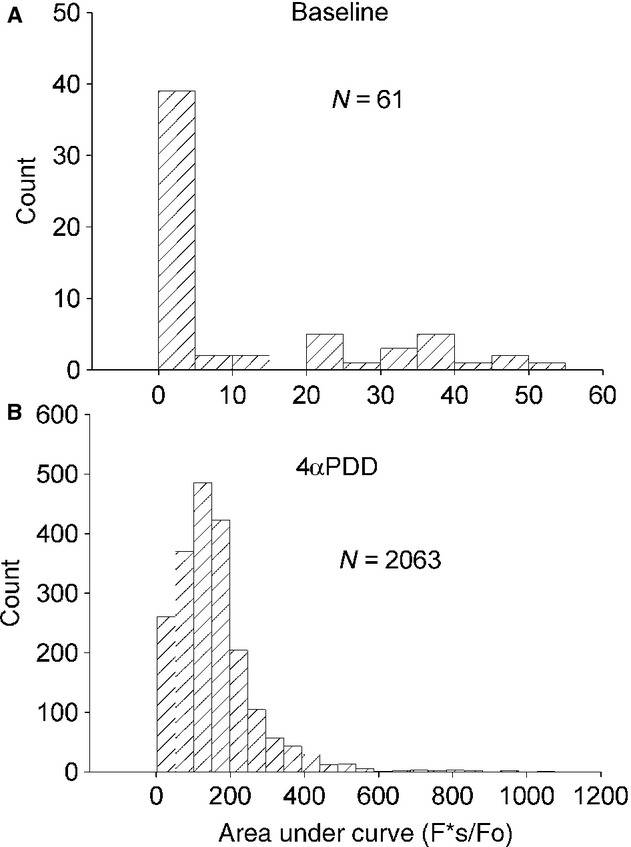
Histograms of the AUC (F*s/Fo) for individual ROI responses in RPMVEC under (A) baseline conditions, and (B) after treatment with 4αPDD.

Figure 3 shows histograms for AUC for all ROI in three monolayers. Figure 3A indicates that 61 cells had calcium activity above the threshold level under baseline conditions, but with much lower durations, amplitude and AUC compared to treated cells. Figure 3B shows the effect of stimulation with 4αPDD, indicating many responding cells (2063) with much greater total calcium activities (AUC) after the drug treatment. Only ∼3% of the cells exhibited baseline calcium activity sufficient to reach threshold levels and these were low-level bursts of short duration compared to the 4αPDD treated cells.

### MLCK inhibition attenuates 4αPDD calcium entry responses

**Figure 4:**
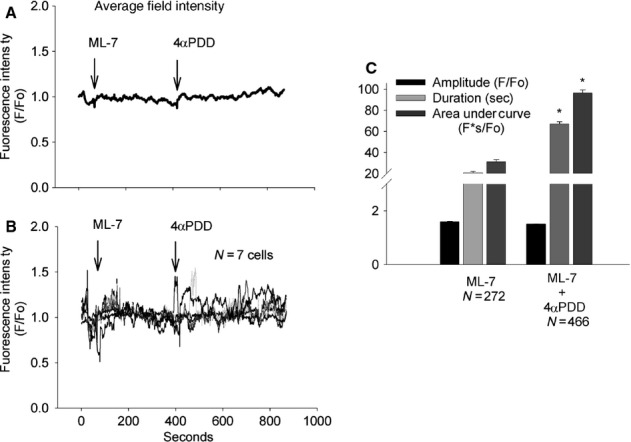
Fluorescence intensities relative to baseline (F/Fo) showing (A) mean monolayer intensities (*N* = 3) during baseline, and treatment with 1 μmol ML-7, followed by 10 μmol 4αPDD, (B) individual ROI responses (*N* = 7) to ML-7 followed by 4αPDD, and (C) means of individual ROI amplitudes, duration, and AUC for the same three monolayers. *P < 0.05 versus ML-7 alone for the same parameter.

Figure 4A shows the average of the mean monolayer intensities of three experiments on RPMVEC pretreated with ML-7 and followed by 4αPDD. There was no significant increase in the mean intensity of the calcium signal immediately following the administration of each drug although the mean calcium tended to rise toward the end of the experiments. Figure 4B shows the fluorescent intensity of seven individual cells indicating that low-level calcium oscillations were increased in some cells after 4αPDD especially toward the end of the experiment.

The LCPro analysis of individual RPMVEC reaching threshold activity in three monolayers after treatment with ML-7 (272) and then 4αPDD (466) are shown in Figure 4C. No significant differences in peak amplitude were determined but mean duration and AUC were significantly higher after 4αPDD, likely due to the greater number of responding cells reaching threshold intensity after 4αPDD.

**Figure 5:**
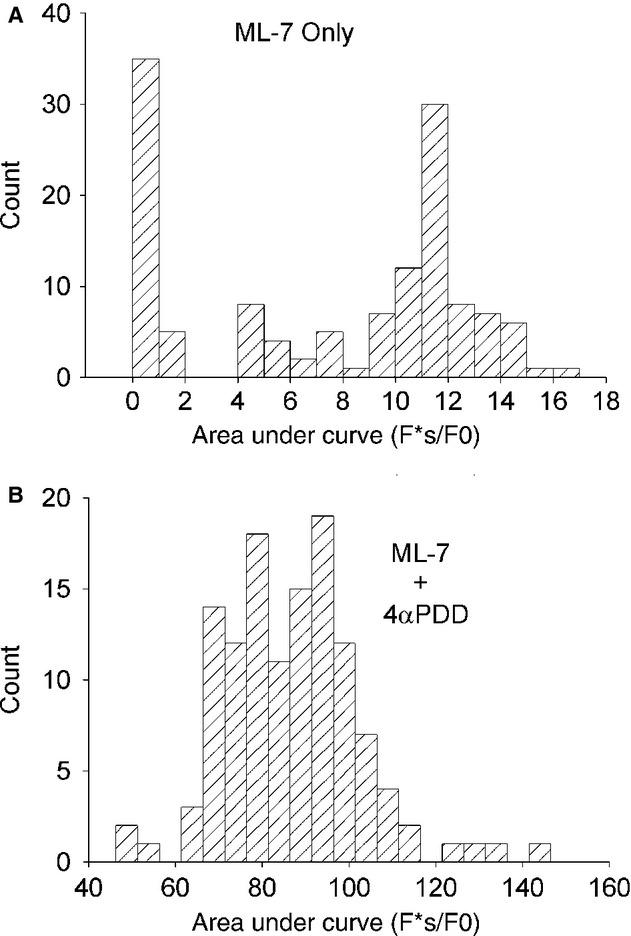
Histograms of individual ROI areas under the curve responses to treatment with (A) ML-7 alone (*N* = 272) and (B) ML-7 followed by 4αPDD (*N* = 466).

Figure 5 is a frequency analysis histogram of AUC in RPMVEC after treatment with (A) ML-7 alone and (B) ML-7 followed by 4αPDD. A detectable increase in total calcium activity occurred after 4αPDD as indicated by AUC in spite of relatively insignificant increases in peak intensity.

**Figure 6:**
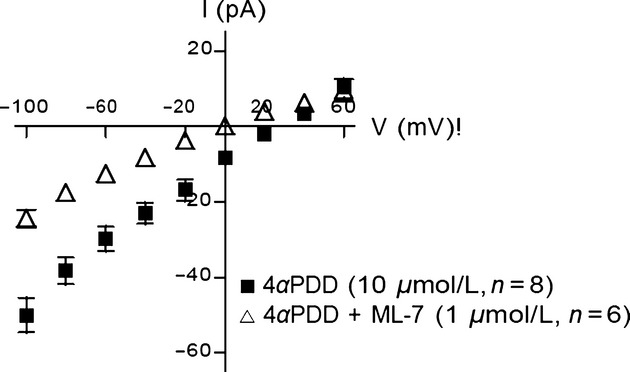
Voltage-current plot in whole-cell patch clamp preparations of individual RPMVEC showing inward currents after treatment with 4αPDD alone, or ML-7 followed by 4αPDD. Inward current was significantly greater (*P* < 0.05) with 4αPDD alone compared to ML-7 followed by 4αPDD.

Calcium entry was also attenuated by ML-7 pretreatment in whole-cell patch clamp measurements of RPMVEC in response to 4αPDD (Fig. 6). The inward calcium currents were significantly diminished by pretreatment with ML-7 (*P* < 0.05). At −100 mV the measured currents were −50.04 ± 4.59 pA for 4αPDD alone and −24.35 ± 2.39 pA for 4αPDD after ML-7 treatment (*P* < 0.05).

**Figure 7:**
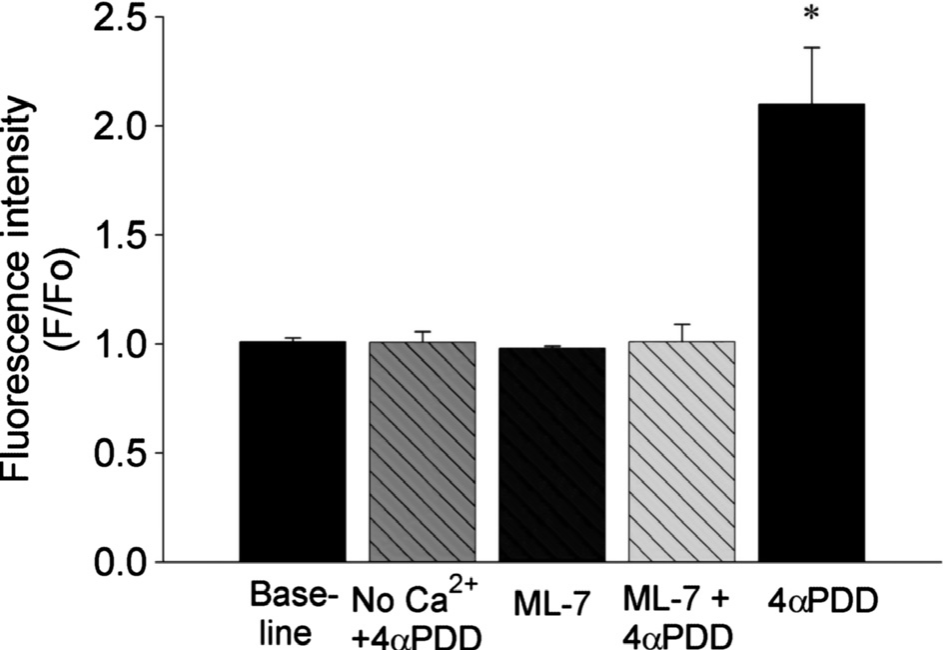
Summary of mean peak monolayer intensities (F/Fo) under baseline conditions, 4αPDD treatment without calcium, ML-7 alone, ML-7 followed by 4αPDD, and 4αPDD alone. *P < 0.05 versus all other groups.

The extracellular source of the intracellular calcium increase with 4αPDD is evident in Figure 7. The average whole field peak intensities for RPMVEC monolayer over the first minute under baseline conditions, after treatment with 4αPDD in calcium-free media, after treatment with ML-7 alone, after treatment with ML-7 followed by 4αPDD, and after treatment with 4αPDD in calcium media are shown. Treatment with 4αPDD alone with calcium present produced a significant increase in the peak whole field intensity of approximately twofold which was significantly higher than that in all other treatment groups, but there were no differences between the mean intensities of the other treatment groups. These studies indicate that the use of only acute changes in whole field monolayer intensity may not reflect subtle and heterogeneous changes in calcium activity of the cell subpopulations.

### ML-7 inhibition of TRPV4 surface protein expression

**Figure 8:**
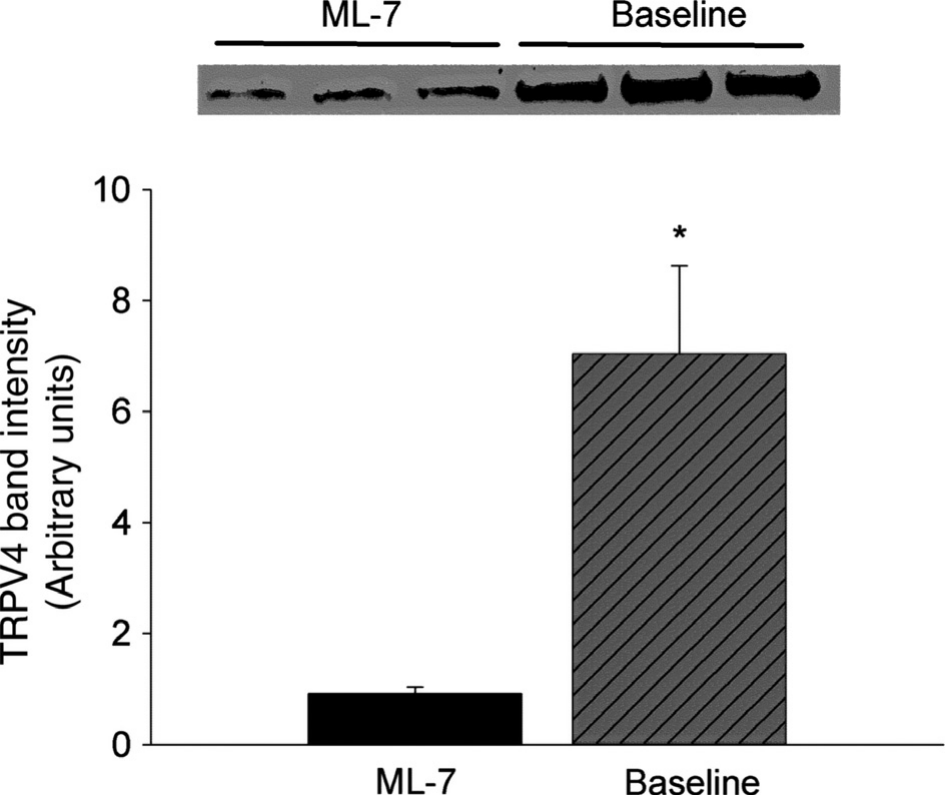
Western blot analysis of surface TRPV4 protein expression in RPMVEC under baseline conditions and after incubation with ML-7. *P < 0.05 versus ML-7 treated.

Figure 8 indicates the surface expression of TRPV4 protein on RPMVEC determined by biotinylation of the surface proteins. The mean of three monolayers indicates an approximately 7.4-fold higher TRPV4 protein in untreated compared to ML-7-treated monolayers. Biotinylation of proteins was performed after separating the cells from the dish, because very little protein was recovered from the apical surface alone.

### Modest effect of dynasore on 4αPDD calcium responses

**Figure 9:**
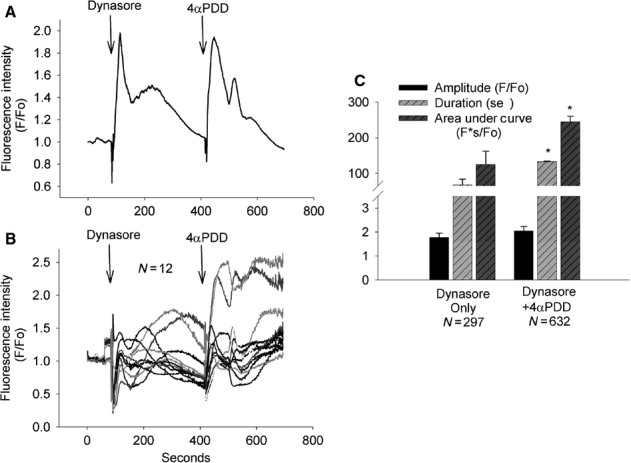
Fluorescence intensities after treatment with dynasore followed by 4αPDD showing (A) mean monolayer intensity, (B) individual ROI responses, and (C) mean amplitude, duration, and AUC for individual ROI's within three monolayers. *P < 0.05 versus dynasore alone for the same parameter.

Dynasore alone produced a robust intracellular calcium increase and amplified the calcium responses to 4αPDD in a subpopulation of endothelial cells. Figure 9 indicates the responses of treating RPMVEC monolayers with dynasore followed by 4αPDD, indicating the (1) mean fluorescent intensity after treatment with dynasore followed by 4αPDD, (2) individual cell ROI's (*N* = 12) showing individual cell responses to dynasore treatment followed by 4αPDD, and (3) a statistical summary of LCPro parameters in three experiments for the amplitude, duration, and AUC of calcium transients after treatment with dynasore alone (297) followed by 4αPDD treatment (632). Duration and AUC were significantly higher for the combination of drugs compared to dynasore alone.

**Figure 10:**
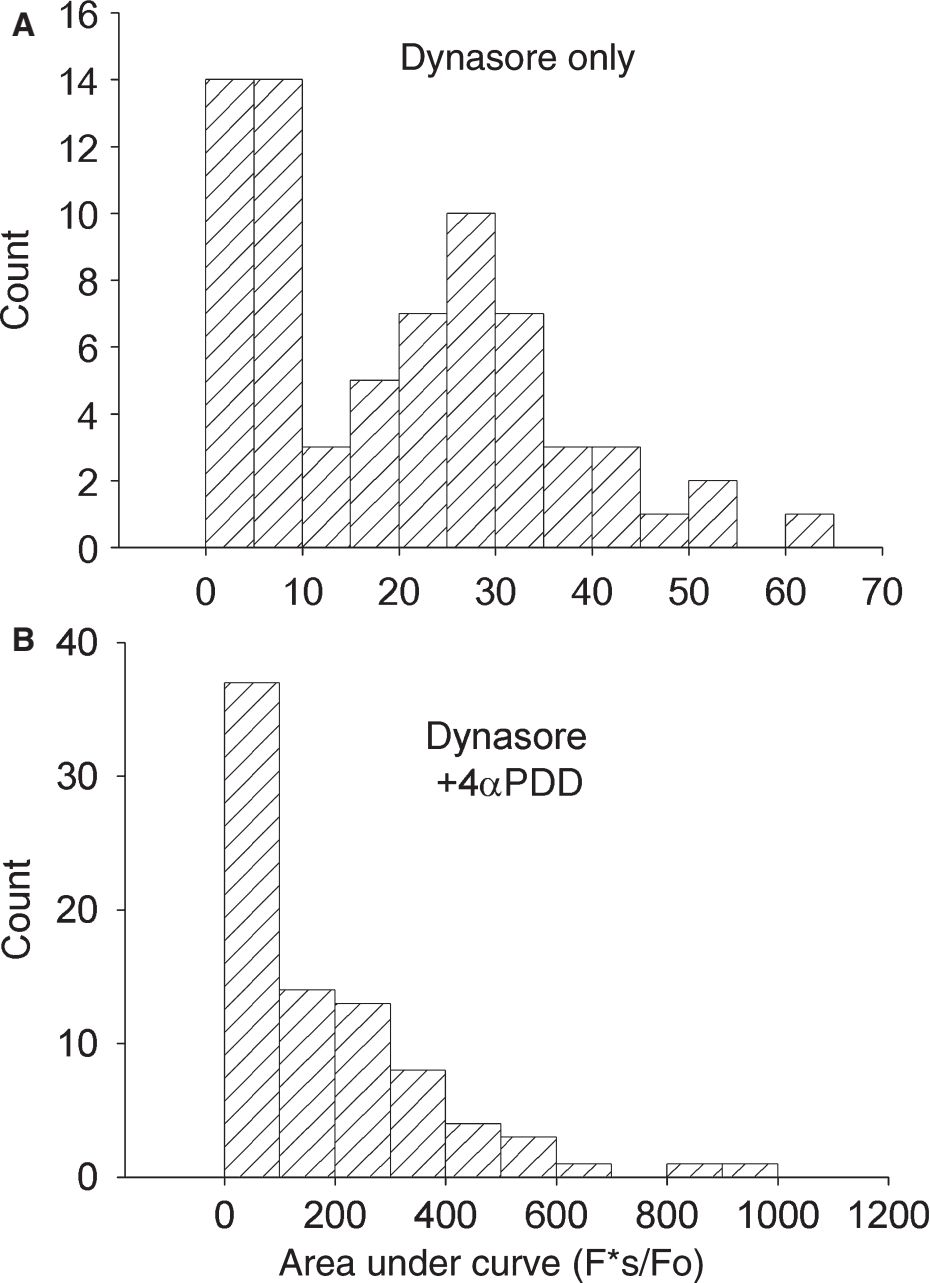
Histograms of AUC for individual ROI's after treatment with (A) dynasore alone, and (B) dynasore followed by 4αPDD.

Figure 10 showing the histograms of the distribution of AUC in RPMVEC monolayers for (1) dynasore alone, and (2) dynasore followed by 4αPDD. The heterogeneous response of cells indicated some cells had a very robust response to 4αPDD following dynasore pretreatment.

### Modest effect of dynasore on TRPV4 surface expression

**Figure 11:**
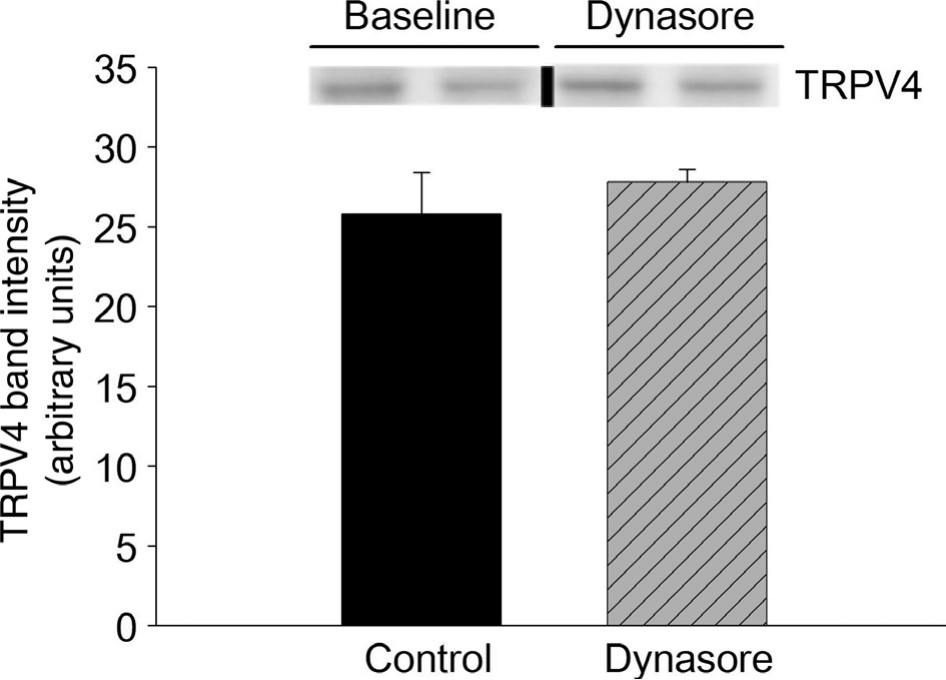
Western blot of surface expression of TRPV4 on RPMVEC monolayers under baseline conditions and after treatment with dynasore. Blots were spiced from the same MW band (vertical black line) and equal loading of samples was confirmed by Ponceau S staining.

As indicated in Figure 11, a Western blot probed for TRPV4 protein after dynasore treatment, the TRPV4 band density at approximately 107 MW was not statistically different from the baseline value. Equal loading of samples was confirmed by Ponceau S staining.

### AUC comparison reveals differential drug responses

**Figure 12:**
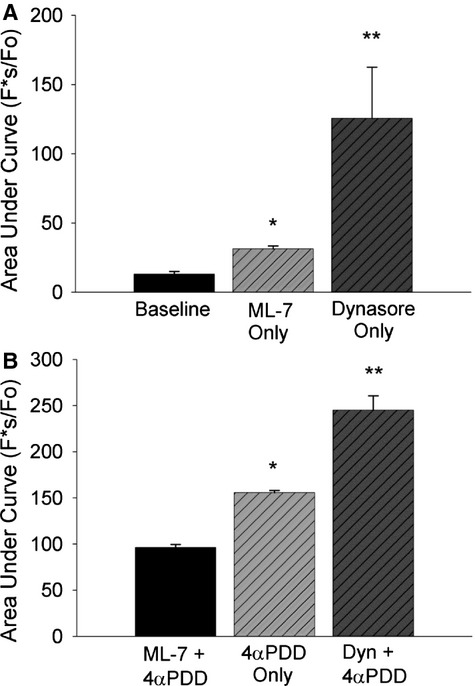
Comparison of AUC for ROI's in all groups showing (A) fluorescence without 4αPDD treatment in cells under baseline conditions, treated with ML-7 alone or dynasore alone; and (B) cells treated with 4αPDD either alone, or following either ML-7, or dynasore pretreatment. (A) *P < 0.05 versus baseline, **P < 0.05 versus all other groups. (B) *P < 0.05 versus ML-7 + 4αPDD, **P < 0.05 versus all other groups.

The AUC measurement demonstrated clear differences between treatments which were not evident using the peak calcium intensities alone. Figure 12 summarizes the AUC for all ROI's detected by LCPro in all treatment groups. Figure 12A shows groups without 4αPDD treatment and indicates that the average AUC were significantly higher than baseline by 4.3-fold after ML-7, or 8.8-fold after dynasore alone. Figure 12B indicates that the average AUC induced by 4αPDD was 36% lower after pretreatment with ML-7 and 57% higher after treatment with dynasore compared to 4αPDD alone. Average AUC after 4αPDD treatment alone was approximately 10.4-fold higher than baseline AUC averages.

### RPMVEC monolayer electrical resistances

**Figure 13:**
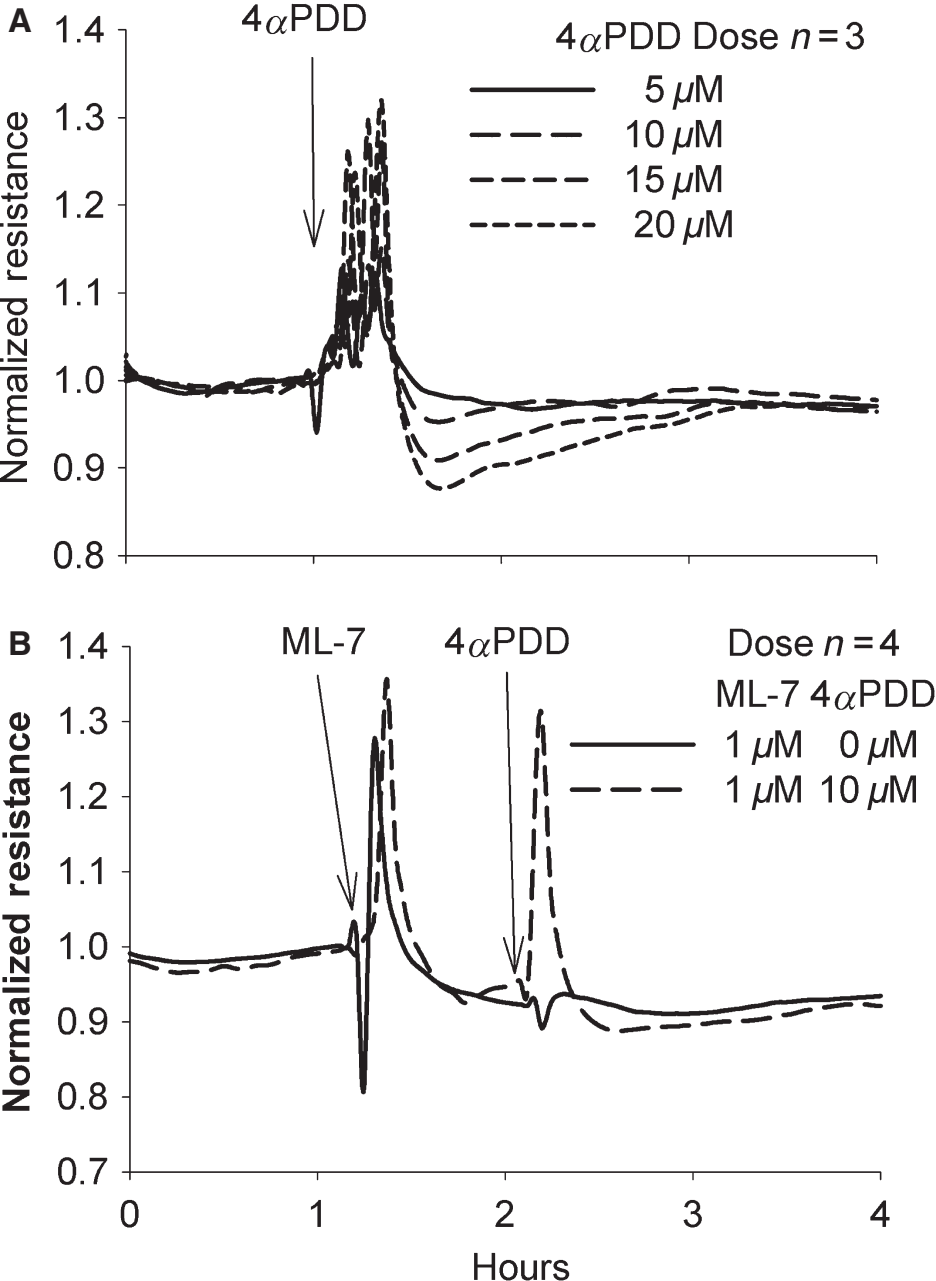
Normalized electrical resistances of RPMVEC monolayers showing (A) 4αPDD dose response curves for doses of 5–20 μmol/L of 4αPDD, (B) 1 μmol/L of ML-7 followed by 10 μmol/L 4αPDD. Each curve represents the average of three experiments.

The electrical resistance responses to 4αPDD of RPMVEC monolayers measured using ECIS are shown in Figure 13. Figure 13A indicates the dose response for 4αPDD doses of 5, 10, 15, and 20 μmol/L. The transient spike increase in resistance represents the resistance artifact during drug application. Each curve represents an average of three experiments. There was a transient resistance decrease that was proportional to the 4αPDD dose with a recovery time which was also inversely related to the 4αPDD dose. The resistance decreases were 0%, 3%, 9%, 13% for the four doses of between 5 and 20 μmol/L. Figure 13B indicates that after pretreatment of the monolayers with 1 μmol/L ML-7, the 10 μmol/L 4αPDD dose produced a decrease of 3% in electrical resistance similar to the response without ML-7 treatment.

**Figure 14:**
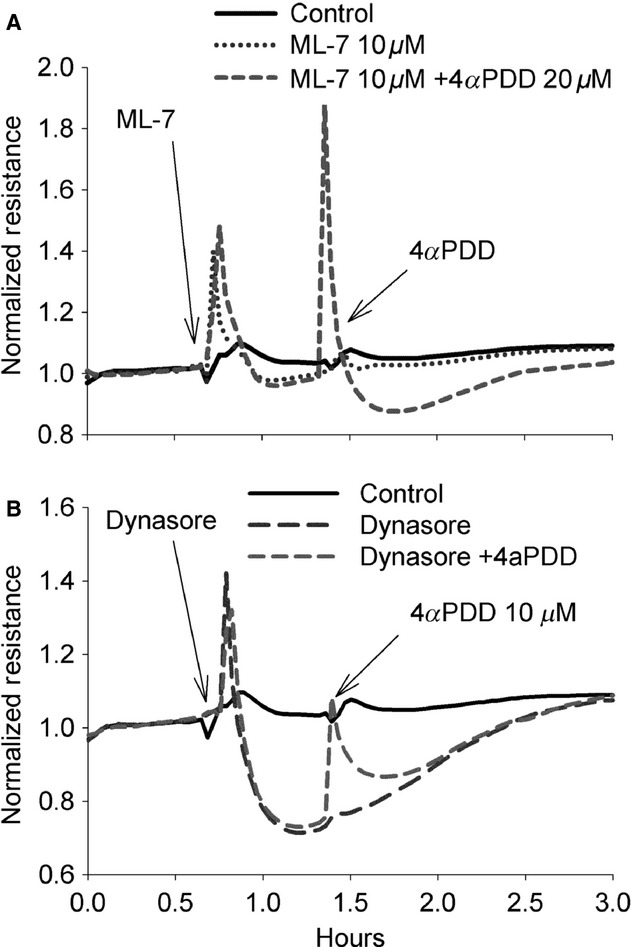
Normalized electrical resistance of RPMVEC monolayers showing (A) 1 μmol/L ML-7 followed by 20 μmol/L 4αPDD, (B) 80 μmol/L dynasore followed by 10 μmol/L 4αPDD. Each curve represents the average of three experiments.

Pretreatment with 1 μmol/L ML-7 followed by a dose of 20 μmol/L 4αPDD produced a resistance decreased of 13% indicating there was little acute effect of ML-7 pretreatment on the resistance effect of 4αPDD within the time frame of these experiments. Figure 14B indicates that treatment with 80 μmol/L dynasore followed by 10 μmol/L 4αPDD produced a monolayer resistance decreased of 29% after the dynasore alone but the 10 μmol/L 4αPDD produced no additional decrease in resistance.

## Discussion

The role of nonmuscle MLCK has been extensively studied in relationship with control of endothelial permeability in response to various receptor-bound mediators and more recently in relation to mechanical stress (Garcia and Schaphorst 1995; Verin et al. 1998; Garcia et al. 1999; Birukov et al. 2002; Dudek et al. 2002). An increased intracellular calcium due to calcium entry or store release can activate calmodulin and subsequently MLCK (Verin et al. 1998). The effects of MLCK activation in inducing endothelial permeability has been interpreted as primarily due to phosphorylation of myosin light chains to increase actin-myosin cell contraction, cytoskeletal remodeling, and opening of paracellular junctions (Birukov et al. 2002). Intracellular calcium may also increase MLC phosphorylation through Rho activation and inhibition of MLC phosphatase (Birukov et al. 2002).

Nonmuscle MLCK has been implicated in both VILI and LPS-induced lung injury (Mirzapoiazova et al. 2011). Inhibition of MLCK with 500 nmol/L ML-7 attenuated the increased filtration coefficient induced by high airway pressure ventilation in isolated perfused lungs (Parker 2000). ML-7, is a cell-permeable inhibitor of MLCK (Ki = 300 nmol/L) which also inhibits PKA and PKC but only at concentrations two log doses higher than those used in this study. Therefore, the 1 μmol/L dose used here would produce inhibition of MLCK without significant effects on other kinases. Subsequent studies by Wainwright and colleagues (2003) demonstrated protection against both VILI and LPS lung injury in non-muscle MLCK knockout mice (Wainwright et al. 2003; Rossi et al. 2007). Mirzapoiazova et al. (2011) confirmed that VILI and LPS injury were also attenuated using either an inhibitory MLCK peptide or inhibitory siRNA for nonmuscle MLCK. However, we show here that inhibition of MLCK can actually reduce calcium entry in response to the TRPV4 agonist, 4αPDD, by reduction in surface expression of the TRPV4 channel protein. In contrast, inhibition of dynamin with dynasore produced a calcium transient itself and also enhanced the subsequent 4αPDD calcium transient in RPMVEC. On the basis of these results, we propose that MLCK may also mediate mechanical lung injury at a point upstream from the myosin actin cytoskeletal effects through control of surface expression of mechanogated cation channels.

Additional support for this concept may be derived from studies of Maniatis et al. (2012) who recently reported attenuation of VILI-induced increases in lung filtration coefficients and albumin permeability, as well as decreases in BAL neutrophils, IL-6 and KC cytokines in caveolin-1 knockout mice. Caveolin-1 and lipid rafts may be involved in vesicular transport of ion channels. Caveolin-1 has a critical role in formation of caveolae as indicated by the almost complete lack of caveolae in endothelium of caveolin-1 knockout mice (Schubert et al. 2001). Plasma membrane lipid rafts also are necessary as cholesterol depletion also interferes with caveolae formation (Lajoie and Nabi 2010). Among the signaling pathways attributed to caveolin-1 and caveolae is the mechanical sensing of hydrostatic pressure and sheer stress (Parton and Simons 2007). The mechanism of mechanical transduction and calcium entry during mechanical stress mediated by caveolin-1 is not known but may involve transport of mechanically sensitive ion channels as suggested by this study.

The overall activity of several types of ion channels is controlled by trafficking of channel containing vesicles to and from the plasma membrane. Some channels are maintained as intact vesicles within the cytoplasm and then recycled to the surface plasma membrane whereas other types are degraded (Bezzerides et al. 2004). Exocytosis of many channel containing vesicles is controlled by myosin motor protein transport to the cell surface whereas endocytosis of channel containing vesicles can be controlled by dynamin which pinches off vesicles during internalization (Eichler et al. 2006). Previous studies have implicated vesicular shuttling of store-operated calcium channels to plasma membranes. MLCK inhibition in RPAECs produced a small release of calcium from internal stores but blocked subsequent thapsigargin-induced calcium entry (Norwood et al. 1999, 2000). Wu et al. (2007) reported that disruption of dynein motor – microtubule system which transports internalized vesicles from the membrane enhanced the inward Isoc current in RPMVEC after thapsigargin. This current is attributed to TRPC1 and TRPC4 expression and is generally not observed in RPMVEC (Wu et al. 2007). Expression of the nonselective store operated channel, TRPC5, also appears regulated by vesicular transport because inhibition of either calcium calmodulin or MLCK decreased calcium entry in response to carbachol, as well as the surface expression of the TRPC5 channel (Kim et al. 2006). Surface TRPC5 channels were also shown to be internalized in a dynamin and clathrin-dependent manner (van de Graaf et al. 2008). In addition, a TRPC1-TRPV4 heteromeric channel has also recently been identified (Ma et al. 2011). Finally, the constitutively active, highly calcium selective TRPV5 and TRPV6 channels are vital for calcium homeostasis and their overall activity is largely regulated by vesicular shuttling to and from the cell membrane (Cuajungco et al. 2006; van de Graaf et al. 2008).

We show here that inhibition of MLCK reduced both surface expression of TRPV4 protein and the calcium response to the TRPV4 agonist in RPMVEC. Previous studies have also directly implicated MLCK in vesicular shuttling of TRPV4 to the cell membrane in other cell types. Masuyama et al. (2012) observed that TRPV4 activity was abrogated by silencing the myosin IIa gene in osteoclasts as well as a reciprocal relation between TRPV4 and calcium calmodulin activity. Strain-induced calcium entry was also inhibited in an alveolar epithelial cell line by inhibition of actin/myosin as well as inhibition of integrin binding (Fois et al. 2012). To enhance TRPV4 surface activity we used dynasore, an inhibitor of the GTPase activity of dynamin that is essential for pinching off clathrin-dependent vesicles formed during endocytosis (Kirchhausen et al. 2008). In this study, an increased surface expression of calcium permeable channels undoubtedly accounted for the increased intracellular calcium and reduced monolayer electrical resistance after dynasore alone. The significantly higher duration and AUC for 4αPDD treatment after dynasore compared to 4αPDD alone suggests increased availability of TRPV4 channels in a subpopulation of the cells even though a significant increase in TRPV4 protein was not detected after dynasore for the monolayer. In HEK cells expressing TRPV4, Cuajungco et al. (2006) reported a 20% increase in the number of cells showing an increased surface TRPV4 expression after dynasore treatment, and overexpression of the dynamin inhibitory protein, PACSIN 3, also increased membrane expression of the TRPV4 channel (Cuajungco et al. 2006). However, the biotinylation method used here may not have been sensitive enough to quantify these small changes in channel abundance, or the biotinylated complex may not bind as readily through the vesicular neck of vesicles which may form but are not pinched off due to dynamin inhibition (Macia et al. 2006).

Analysis of the 4αPDD calcium transients in individual cells using the LCPro analysis resulted in a more sensitive analysis of these calcium transients compared to using whole field peak intensities alone because highly significant differences were obtained between groups for duration and AUC when there was little or no difference between peak intensities for the groups. The total 4αPDD calcium activity indicated by curve duration and AUC were significantly increased with dynasore and significantly decreased with ML-7 (Francis et al. 2012). The AUC response to 4αPDD after dynasore appeared to be heavily weighted by a subpopulation of endothelial cells, suggesting a heterogeneous distribution of the TRPV4 channels.

The relatively modest monolayer resistance decrease induced by 4αPDD in RPMVEC was expected as pulmonary microvascular endothelium is much less responsive to permeability agonists than endothelium from other lung vascular segments (Stevens 2011). However, the apparent inability of MLCK inhibition to abrogate the resistance decrease due to 20 μmol/L 4αPDD, or of dynasore to enhance the resistance decrease induced by 4αPDD was unexpected based on previous studies in intact lungs (Alvarez et al. 2006). Activation of macrophages and other lung cell types likely accounts for these differences in responses between cultured RPMVEC monolayers and intact lungs (Hamanaka et al. 2007), because VILI is dependent upon calcium entry through stretch activated cation channels in all lung vascular segments and cell types (Parker et al. 1998; Parker and Yoshikawa 2002).Hamanaka et al. (2007) identified the TRPV4 channel as the essential mechanogated channels in VILI, because VILI in isolate mouse lungs was attenuated by pretreatment either with inhibitors of TRPV4 (ruthenium red), arachidonic acid production (methanandamide), or P-450 epoxygenases (miconazole), or TRPV4 gene product deletion, whereas injury was augmented at higher temperatures (Hamanaka et al. 2007). In previous studies, VILI was also attenuated by inhibition of phospholipase A2, a major source of stretch-induced arachidonic acid known to gate the TRPV4 channel (Yoshikawa et al. 2005; Miyahara et al. 2008). Macrophages activation has also been implicated as an essential step in VILI because Hamanaka et al. (2010) found that wild type macrophages instilled in lungs of TRPV4 knockout mice restored the increased permeability response to mechanical injury, and other studies reported that deletion of alveolar macrophages attenuated VILI (Frank et al. 2006; Eyal et al. 2007). Inhibition of MLCK inhibited store-operated calcium entry in lung macrophages and stable binding of MLCK to macrophage migration inhibitory factor (MIF) in endothelial cells also suggests involvement of MLCK in macrophages activation (Tran et al. 2001). In addition, priming of microvascular endothelial cells by macrophages mediators may be necessary to develop the full microvascular permeability response to mechanical injury as previously demonstrated for other permeability agonists (Wang et al. 2008).

In summary, these studies suggest that mechanical injury mediated by TRPV4 channels may be regulated to a great extent by vesicular shuttle activity which modulates the channel density on the plasma membrane. The MLCK inhibitor, ML-7, greatly reduced intracellular calcium entry after the TRPV4 agonist 4αPDD, whereas the overall calcium entry response to 4αPDD was enhanced by the dynamin inhibitor, dynasore. Surface expression of biotinylated TRPV4 was reduced by ML-7 but we could not detect a statistically significant change after dynasore. 4αPDD in the dose used for these experiments produced little decrease in monolayer resistance, and the resistance decrease induced by dynasore was not enhanced by 4αPDD. The relative insensitivity of the microvascular monolayer electrical resistance to 4αPDD and inability of ML-7 to block the high-dose 4αPDD resistance decrease suggest that interaction the vascular endothelium with other lung cells such as the macrophages are essential for regulating vascular permeability in the intact lung.

## Supporting Information

**Video S1.** RPMVEC baseline calcium activity.

**Video S2.** RPMVEC 4aPDD Calcium response.

**Video S3.** RPMVEC ML-7 & 4aPDD responses.

**Video S4.** RPMVEC dynasore & 4aPDD responses.
